# Diagnosis of *Hymenoptera* Venom Allergy: State of the Art, Challenges, and Perspectives

**DOI:** 10.3390/biomedicines10092170

**Published:** 2022-09-02

**Authors:** Joanna Matysiak, Eliza Matuszewska, Kacper Packi, Agnieszka Klupczyńska-Gabryszak

**Affiliations:** 1 Faculty of Health Sciences, Calisia University-Kalisz, 62-800 Kalisz, Poland; 2 Department of Inorganic and Analytical Chemistry, Poznan University of Medical Sciences, 60-806 Poznan, Poland; eliza.matuszewska@ump.edu.pl (E.M.); kacperpacki1@wp.pl (K.P.); aklupczynska@ump.edu.pl (A.K.-G.); 3 AllerGen, Center of Personalized Medicine, 97-300 Piotrkow Trybunalski, Poland

**Keywords:** *Hymenoptera* venom allergy, diagnostics, *Hymenoptera* venom allergens, cross-reactive carbohydrate determinants

## Abstract

*Hymenoptera* venom allergy is the most common cause of anaphylaxis in adults and the second-most frequent in children. The proper diagnosis of this life-threatening allergy remains a challenge. This review focuses on the current knowledge regarding diagnostics of *Hymenoptera* venom allergy. The paper includes a brief description of the representatives of *Hymenoptera* order and the composition of their venoms. Then, diagnostic tests for allergy to *Hymenoptera* venom are described. Common diagnostic problems, especially double positivity in tests for IgE antibodies specific to honeybee and wasp venom, are also discussed. Special attention is paid to the search for new diagnostic capabilities using modern methodologies. Multidimensional molecular analysis offers an opportunity to characterize changes in body fluids associated with *Hymenoptera* venom allergy and yields a unique insight into the cell status. Despite recent developments in the diagnostics of *Hymenoptera* venom allergy, new testing methodologies are still needed to answer questions and doubts we have.

## 1. Introduction

**Figure 1:**
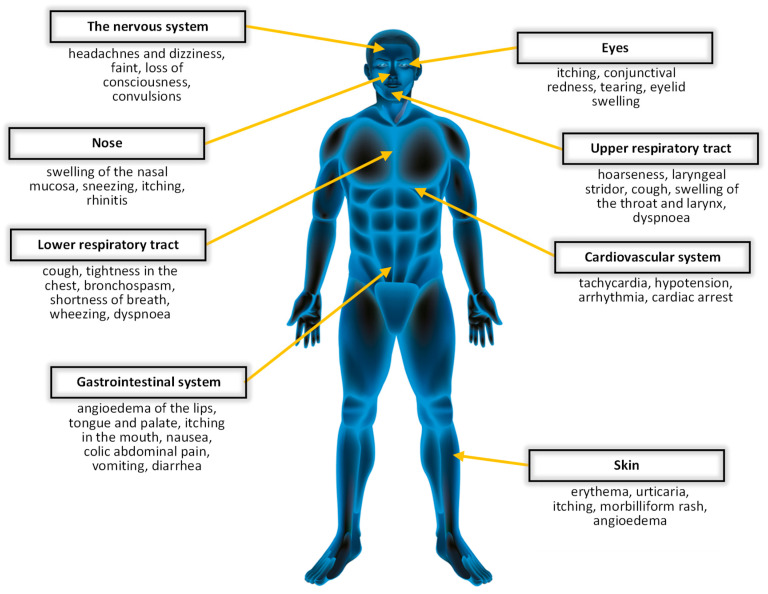
Allergic symptoms following a *Hymenoptera* sting.

*Hymenoptera* venom allergy is the most common cause of anaphylaxis in adults and the second-most frequent in children [1,2,3]. After an insect sting, normal local reaction (NR), large local reaction (LLR), or a whole spectrum of general symptoms, including anaphylactic shock, may occur. According to European data, the frequency of systemic reactions ranges from 0.3 to 8.9% in adults and even up to 32% in beekeepers [2]. Systemic symptoms may affect the skin (itching, urticaria, angioedema), respiratory (stridor, dyspnea, cough, bronchial obstruction), cardiovascular (hypotension, tachycardia, arrhythmias, cardiac arrest), gastrointestinal (vomiting, stomach pain, diarrhea), or even nervous system (dizziness, fear of death, fainting)—Figure 1 [4]. Fatal reactions are rare [5], but *Hymenoptera* stings cause 20% of cases of anaphylaxis-related fatalities [6]. *Hymenoptera* venom allergy is therefore a life-threatening allergy, and all patients who develop systemic symptoms after the sting require a detailed diagnosis. Proper diagnostics allows for the appropriate management and implementation of venom immunotherapy (VIT) [7,8,9]. VIT is currently the best form of treatment for venom allergies because it reduces the risk of subsequent severe reactions. It is also a therapy that significantly improves the quality of life. It should be emphasized that the proper qualification for VIT and its effectiveness depends on the correct diagnosis. Today, we know better and have better diagnostic tools, thanks to which diagnostics and therapy can be individualized to the needs of a single patient [10].

### 1.1. Hymenoptera

**Figure 2:**
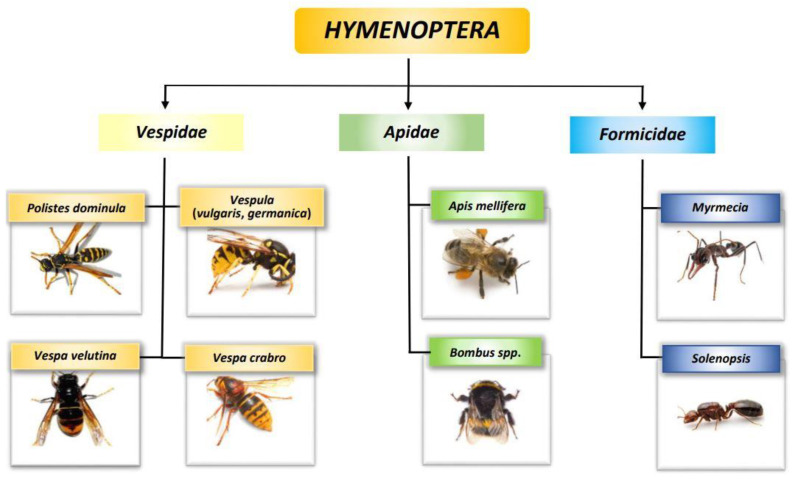
Taxonomy of *Hymenoptera*.

The basic element in the diagnosis of venom allergy is the identification of the insect that has stung the patient—Figure 2. It is not only patients that have problems with distinguishing *Hymenoptera* insects, but sometimes also doctors [11,12]. Therefore, it is worth knowing the taxonomy of *Hymenoptera* insects and identifying their appearance and characteristics. Most allergic reactions after stings in Europe are caused by insects of the *Apidae* and *Vespidae* families [13], and very rarely they are caused by *Formicidae*—these are often present in America (red fire ant), Australia (jumper ant), and Asia (Asian needle ant)—Figure 2 [14]. The *Vespidae* family includes *Vespula* spp. (*Vespula vulgaris, Vespula germanica*)—named wasps in and yellow jackets, and hornets (*Vespa crabro*) and paper wasps, (*Polistes dominula*) in Europe and the USA, respectively. The most important representative of the *Apidae* family is the honeybee (*Apis mellifera*) and the less frequently stinging bumblebee (*Bombus terrestris, B. agrarum, B. medics*) [15]. The honeybee is the most aggressive towards humans. By contrast, wasps sting most often because they feed on sugars and are attracted to human food and drink, especially outdoors. In terms of allergy, hornets can be treated like large wasps. They are scary due to their size. However, they are relatively non-aggressive insects—they sting only when threatened, e.g., when we breach their nest. Bumblebees cause anaphylaxis mainly in gardeners since their role is to pollinate. Particularly noteworthy is the species *Vespa velutina*. At the beginning of the first decade of the 21st century, it was imported from China and began to spread through Western Europe. Contrary to media reports, scientific data from Europe show that this insect does not show more aggression towards humans than the other *Vespidae*, and the effects of its stings are similar to those of native species [16,17,18].

### 1.2. Hymenoptera Venoms

**Figure 3:**
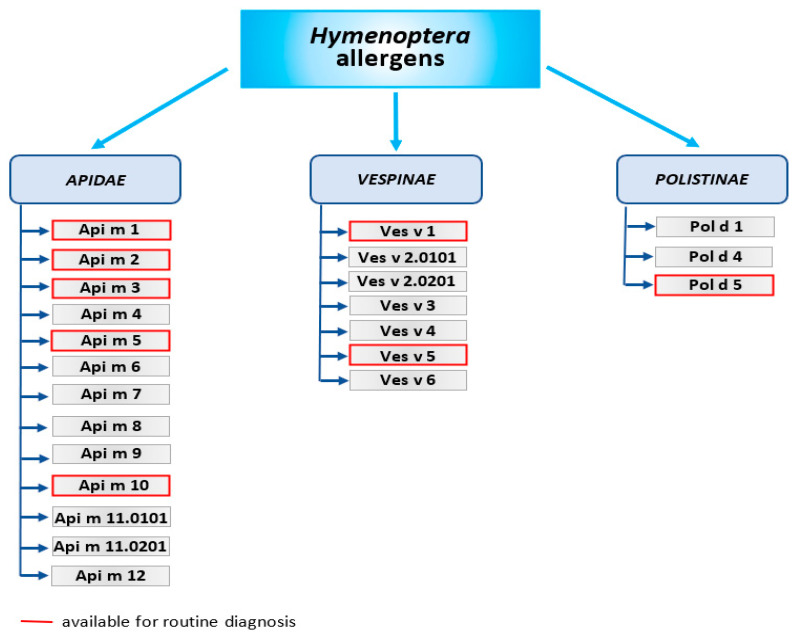
Allergens of *Hymenoptera* venoms.

Venoms of *Hymenoptera* are secreted by glands located in the abdominal cavity of insects. Bees and other species use venom mainly for defense against predators. Hence, *Hymenoptera* venoms are complex mixtures of different bioactive molecules that affect the intruder’s body. These odorless, transparent, and acidic liquids consist of biogenic amines, basic peptides and proteins—most of them have enzymatic activity [19]. Moreover, venoms contain sugars, phospholipids, pheromones, volatile compounds, and are also made up of about 80% water [20]. The best examined *Hymenoptera* venom so far is honeybee venom. Although several hundred proteins and peptides contained in bee venom have already been identified, its full composition is still not characterized [21,22]. So far, at least 18 compounds of *Apis mellifera* venom have been reported to have biological activity [23]. Among these are 12 allergens that are responsible for the allergic reactions that occur after exposure to venom. In venoms of other *Hymenoptera* species, five allergens have been identified in venoms of *Vespula vulgaris* and *Polistes dominula*, four allergens in the venom of *Solenopsis invicta*, three allergens in the venom of *Myrmecia pilosula*, and two allergens in venoms of *Vespa crabro* and *Bombus terrestris* (according to www.allergen.com (accessed on 19 July 2022))—Figure 3.

The major *Apidae* venom allergens include [24]:

- Api m 1 is the phospholipase A2—the major allergen of honeybee venom. Phospholipase A2 is also contained in *Bombus* spp. venoms (Bom p 1 and Bom t 1), but the protein sequence identities between *Apis mellifera* and *Bombus* spp. is approximately 52–53%. Venoms of wasps contain phospholipase A1—Ves v 1 and Pol d 1, which differs in sequence and substrate specificity.
- Api m 2—hyaluronidase, the second major allergen of bee venom. Due to the similarity structure of bee hyaluronidase and wasp hyaluronidase, this allergen is the main cause of cross-reactions of IgE antibodies against the venom of these insects. Wasp hyaluronidase exists in 2 isoforms—Ves v 2.0101 and Ves v 2.0201.
- Api m 3—acid phosphatase is an enzyme that catalyzes the hydrolysis of monoesters and anhydrides of phosphoric acid. Together with the Api m 1 and Api m 2, it is considered as one of the most potent venom allergens.
- Api m 4—melittin is a minor allergen but the main component of bee venom, accounting for about half (45–50%) of its dry weight. Melittin possesses strong hemolytic and antimicrobial properties. Api m 3 and Api m 4 have been identified only in bee venom so far.
- Api m 5—dipeptidyl peptidase-IV and its analogues (Ves v 3 and Pol d 3) are present in wasp venom and the European paper wasp venom. Ves v 3 and Pol d 3 are major allergens that show high cross-reactivity.
- Antigen 5—an allergen found in the venom of almost all *Vespoidea* insects; Ves v 5 is found in the venom of yellow jackets; and Pol d 5 is found in the venom of paper wasps. Its function is still unknown. However, it is known to be highly allergenic and responsible for cross-reactions. Antigen 5 is a key element in CRD (component-resolved diagnostics) to distinguish between bee and wasp venom allergy [25].

Other *Apidae* venom allergens are minor allergens:

- Api m 6—putative protease inhibitor. It stands for only 1–2% of venom’s dry weight and is considered one of the weakest bee venom allergens.
- Api m 7—CUB (Clr/Cls, urchin EGF-like protein and bone morphogenic protein 1) serine protease, which shows high IgE-binding activity.
- Api m 8—carboxylesterase-6. Bee venom contains less than 1% of this allergen, and its function is not yet fully elucidated.
- Api m 9—venom serine carboxypeptidase that belongs to the peptidase S10 family. There are no reports on the immunogenic properties of Api m 9.
- Api m 10—icarpin. This carbohydrate-rich protein is identified as a key allergen due to its importance not only in the diagnosis of bee venom allergy, but also in immunotherapy. VIT may be ineffective in patients allergic to Api m 10 due to being underrepresented in some therapeutic extracts [26].
- Api m 11—occurs in two isoforms: major royal jelly protein (MRJP) 8 (Api m 11.0101a), and MRJP9 (Api m 11.0201a). MRJPs are a family of proteins identified only in the *Apis* spp. These proteins constitute about 90% of all royal jelly (RJ) proteins. Human IgE antibodies recognize MRJP1 present in the sera of patients allergic to RJ, as well as to bee venom.
- Api m 12—vitellogenin, 200 kDa peptide belonging to the vitellogenin family and cross-reactive to Ves v 6.

Among allergens of the *Vespinae* and *Polistinae*, we distinguish:

- Ves v 1 and Pol d 1—phospholipase A1, a peptide with hemolytic activity.
- Ves v 2 and Pol d 2—hyaluronidase (in *Vespinae* isoforms Ves v 2.0101, Ves v 2.0201 exist) hydrolyzes hyaluronic acid. This may cause a pathogenic reaction in allergic patients.
- Ves v 3 and Pol d 3—dipeptidyl peptidase-IV, peptides with high cross-reactivity, resulting from 76.1% sequence identity. Their function is activating or inactivating substrates by cleaving dipeptides.
- Ves v 5 and Pol d 5—antigen 5, considered the most important wasp allergen, but its function is still unknown.
- Ves v 6—vitellogenin, considered an IgE sensitizer.

## 2. Diagnosis of *Hymenoptera* Venom Allergy

Diagnostic tests for allergy to *Hymenoptera* venom should be performed in all patients with a history of systemic reaction following a *Hymenoptera* sting. Tests should also be performed optionally in patients with a history of LLR if they have a particularly high risk of being re-stung, e.g., beekeepers, farmers etc., and have a significant reduction in quality of life because of this [27,28,29].

The diagnosis of *Hymenoptera* venom allergy in all the abovementioned cases yields qualification for VIT. Immunotherapy is the most effective method of allergy treatment, during which the immune system develops an immunological tolerance to the allergen. However, it is a time-consuming method (3–5 years of therapy) and is associated with a risk of some side effects. That is why making the right diagnosis and starting VIT in a patient is so important.

**Figure 4:**
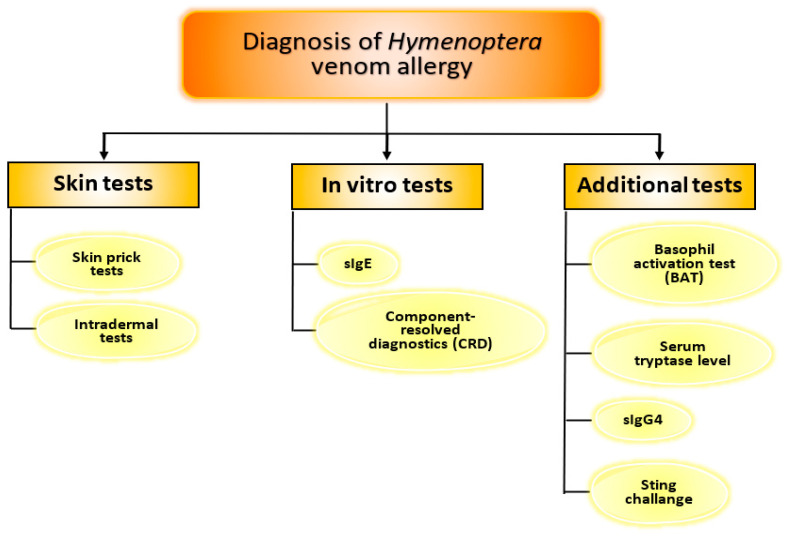
Diagnostic tools in *Hymenoptera* venom allergy.

Routine diagnostic tests include skin prick tests (SPTs) and venom sIgE level determination—Figure 4. To avoid false negative results, the mentioned tests should be performed at least 2–4 weeks after the sting (the so-called refractory period) [30]. According to European guidelines, SPTs should be performed first. If their results are negative, the next step involves intradermal tests [30,31]. Intradermal tests have a much higher sensitivity than prick tests, which has been shown in clinical studies. SPTs identify 49% of patients with *Hymenoptera* venom allergy (HVA), whereas the combination of SPTs and intradermal tests made it possible to diagnose 94% of patients [32]. In Europe, standardized venoms of *Apis mellifera, Vespula* spp., *Polistes* spp. and *Vespa crabro* are available and can be used for skin tests. Moreover, skin tests have been shown to be safe, even in patients with mastocytosis [33,34].

The disadvantage of skin tests is that they are performed with venom extracts that may contain too little or even no allergen components at all. For this reason, they may be negative in patients only allergic to selected allergen proteins, e.g., Api m 10 [35]. Another problem with skin testing is the frequent double sensitizations with whole extracts.

Determination of venom sIgE with whole venom extracts has lower sensitivity than skin tests—even 20% of patients with positive skin tests have negative in vitro tests to whole extracts of venoms. Meanwhile, only 10% of patients with negative skin tests have positive venom sIgE. For this reason, it is currently recommended to perform skin tests and serological tests together [36]. In addition, it was observed that the sensitivity of wasp venom sIgE ranges from 83% up to 97% and is lower than for honeybee venom sIgE—98–100% [37].

The much higher sensitivity of in vitro tests is obtained by performing a component-resolved diagnosis (CRD) [38,39,40]. This innovative method is an increasingly better diagnostic tool in allergology today. Thanks to CRD, sIgE against single allergenic proteins can be determined. Using CRD, primary sensitization and cross-reactivity in patients with double positive results to whole extracts can be distinguished [41]. Very often (up to 75%), double sensitization to honeybee and yellow jacket venom is due to cross-reactive carbohydrate determinants—CCDs. Currently, allergens for CRD testing are produced as recombinant allergens without the naturally occurring insect glycosylation (CCDs).

Commercially available *Hymenoptera* allergens for CRD are:

- rApi m 1, rApi m 2, rApi m 3, rApi m 5, rApi m 10;
- rVes v 1, rVes v 5;
- rPol d 5.

All abovementioned recombinant allergens are in CCD-free form.

Using the diagnostic capabilities of CRD, the sensitivity of tests for bee venom allergy reaches 94.4% [42]. To achieve the sensitivity of diagnostic tests of 94–95% for allergy to honeybee venom, all six available allergen components—Api m 1–5 and Api 10—should be determined. It was also found that 74% of patients had sIgE to more than one allergen of bee venom. In wasp venom allergy, thanks to the combination of rVes v 5 and rVes v 1 determination, a sensitivity of up to 98% was observed [43].

CRD tools facilitate the differentiation of cross-reactions between bee and wasp venom allergens [44] and are also more sensitive in patients with very low levels of sIgE to whole extracts [45]. In double sensitization, the CRD method is often helpful, but its possibilities are still limited and do not yield a resolve for all ambiguous clinical cases [46].

In the case of allergy to different wasp species (often between *Vespula* and *Polistes*), differential diagnosis is more complicated due to the independence from cross-reactions associated with CCDs [47,48]. This problem certainly requires further research. It may be beneficial to introduce further allergens into routine diagnostics—at least Pol d 1.

The possibilities offered by CRD contribute to the improvement not only of the diagnosis of venom allergy, but also better therapy—especially the appropriate qualification for VIT. So far, there was no correlation found between diagnostic test results (including CRD tests) and the severity of the allergic reaction after a sting [49]. However, some correlations between sIgE to bee venom allergens and the effectiveness of VIT was observed. Sensitization to Api m 3, Api m 5, and Api m 10 is a risk factor for the failure of VIT because of insufficient content of these allergens in therapeutic extracts [26,50]. Besides, as mentioned above, CRD tests are helpful in patients with double sensitization and cross-reactions to different venoms.

## 3. Additional Tests

Among other additional diagnostic methods used in the diagnosis of *Hymenoptera* venom allergy, the following tests should be mentioned: basophil activation test (BAT), measurement of basal serum tryptase (BST) level, and sIgG4—Figure 4.

The BAT is recommended if all other diagnostic tests fail to make a proper diagnosis in patients with a history of systemic reactions to a sting [51]. In the BAT, the whole blood is stimulated with venom allergens, and the activation of basophils is measured by flow cytometry. This test can be performed in highly specialized laboratories, which limits its availability. A total of 67–81% of patients with negative skin tests and in vitro tests have a confirmed diagnosis of venom allergy by using the BAT [51,52]. The BAT is also recommended in the case of patients with a double positive result to *Hymenoptera* venoms and inconclusive results with recombinant allergens.

BST levels should be marked in any case of systemic symptoms after a sting. An elevated BST shows a significantly greater risk of severe reactions to *Hymenoptera* stings. BST is also higher in patients with mastocytosis [53,54].

Venom sIgG4 arises in contact with an antigen and may play a protective role [55]. Significantly higher levels of bee venom sIgG4 were detected in beekeepers, who are regularly stung by bees and do not experience allergic reactions, despite positive venom sIgE determinations. Increasing levels of sIgG4 are also observed during VIT. Further research is required to find out whether they can be used as a marker in VIT.

The sting challenge test with a live insect is currently not recommended as a routine diagnostic method because of the high risk of severe reactions after the sting. However, the sting challenge test with a live insect can be used to evaluate the efficacy of venom immunotherapy, especially in patients with a high risk of re-sting [15,56].

To summarize, it can be stated that modern diagnostics of *Hymenoptera* venom allergy are highly sensitive, especially with the use of CRD methods. By performing skin tests and in vitro tests together and, in special cases, additional tests, it is possible to diagnose most patients. However, it is still not possible to diagnose all patients who develop general symptoms following a sting. As shown, there are still difficulties in the case of double sensitization to venoms and making a decision about appropriate immunotherapy in this case is not easy. Venom allergy is a potentially life-threatening allergy; therefore, making such a diagnosis has a significant impact on the patient’s quality of life. There are always questions about the likelihood of another anaphylactic reaction. While we have more and more improved diagnostic tools, it should be emphasized that the results of skin and serological tests do not correlate with the severity of symptoms after the sting. Therefore, it is necessary to search for even better biomarkers of venom allergy, which can improve the diagnosis and effectiveness of immunotherapy.

## 4. Searching for New Diagnostic Capabilities of *Hymenoptera* Venom Allergy

Studies conducted to determine the protein–peptide profile in biological material obtained from allergic patients and healthy individuals (control group) have shown a relationship between the presence of a condition and the composition of body fluids/tissues. Molecular differences between pathological and normal profiles can be used as biomarkers of disease processes and serve as a tool for correctly diagnosing and treating allergic conditions [57,58,59,60,61].

Proteins whose levels in the examined biological material correlate with the allergic disease have a regulatory function. According to the available literature, potential biomarkers include the hornsin, psoriasin, and calgranulin-B, as well as apolipoproteins, particularly apolipoprotein E9 and apolipoprotein H [57,59,60,62]. Acute phase proteins are the second crucial functional group of structures that are characteristic of allergic diseases. Their concentrations change in response to inflammation. This group includes plasma albumin, transferrin, lactoferrin, fibrinogen (alpha chain), and orosomucoid (ORM-1 and ORM-2) [62,63,64].

However, very few experimental reports focus on diagnosing *Hymenoptera* allergy using modern analytical platforms, such as liquid chromatography coupled with mass spectrometry. Our research team has previously proposed a sophisticated methodology for searching for *Hymenoptera* venom allergy markers, based mainly on the mass spectrometry technique. For depicting alterations in protein–peptide patterns caused by bees’ and wasps’ venom, a highly sensitive and specific nano-liquid chromatography—matrix-assisted laser desorption/ionization time-of-flight mass spectrometry (nanoLC-MALDI-TOF MS) method was utilized [65]. As a result of the study, changes caused by venom administration have been observed in several compounds present in human serum. The proteins that change in allergic patients exposed to venom were coagulation factor XIII chain A, fibrinogen alpha chain, complement C4-A, and inter-alpha-trypsin inhibitor heavy chain H4. All these proteins participate in inflammation following an insect sting.

**Figure 5:**
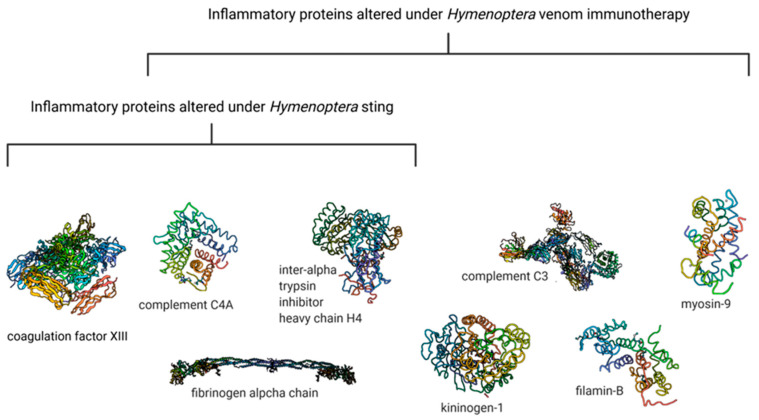
Proteins changed under *Hymenoptera* insect sting and VIT (based on MALDI-TOF analyses).

Additionally, in the previous study performed by our team using the MALDI-TOF MS technique, we identified inflammation factors whose expression were altered in human serum under the influence of *Hymenoptera* venom immunotherapy [66]. The results of the abovementioned research indicate that most of the identified proteins change in serum after occasional stinging by insects and after venom-specific immunotherapy-Figure 5.

Moreover, to discover new markers of *Hymenoptera* venom allergy in a group of inflammation factors, a Bio-Plex (Bio-Rad, Hercules, CA, USA) multiplex immunoassay was utilized [67]. Bio-Plex analyses indicate that levels of three inflammatory factors significantly change after venom exposition. These factors were: soluble CD30/tumor necrosis factor receptor superfamily, member 8 (sCD30/TNFRSF8), soluble tumor necrosis factor receptor 1 (sTNF-R1), and matrix metalloproteinase-3 (MMP-3).

The results of the proteomics studies indicate that *Hymenoptera* venom affects the expression of inflammatory features in serum. According to the available literature, the development of localized and systemic allergic reactions following the *Hymenoptera* sting is related chiefly to allergen-specific IgE antibodies. Consequently, the mediators of inflammation are released to restore homeostasis and neutralize toxins [68,69]. The abovementioned results prove these well-known reports, as several inflammatory features were marked as differentiating between allergy and healthy controls. The findings indicate that as a result of venom exposition, the complement pathway is activated, causing the production of kinin and the symptoms of allergic sensitization. This mechanism is based on various reactions and the involvement of other complement components proteins which differentiate the studied groups, such as fibrinogen and coagulation factors. The inflammation is observed both after spontaneous stings and after controlled administration of small amounts of venom as immunotherapy. Additionally, both sTNFR-1 and sCD30 molecules can be essential in regulating or activating allergic inflammation [70,71,72].

Moreover, for the search of the *Hymenoptera* venom allergy biomarkers, the methodology based on two-dimensional gel electrophoresis (2-DE) and LC–Orbitrap-MS was applied [73]. This study identified a cleaved form of haptoglobin as a specific protein marker in patients allergic to bee venom. Haptoglobin is a member of the family of acute-phase plasma proteins. It acts as a plasma detoxifier. This protein may be potentially used for the diagnosis of *Hymenoptera* allergy. Interestingly, the results suggest that cleaved haptoglobin may be a significant prognostic protein for anaphylaxis. It has been reported that a high incidence of hypersensitivity to bee venom may be associated with the haptoglobin genotype.

The molecular changes in serum caused by *Hymenoptera* venom also affect the metabolic profile. A study performed with liquid chromatography–tandem mass spectrometry revealed almost 300 metabolites that were significantly different among patients stung by wasp versus healthy controls [74]. The investigation of the metabolic profile indicated that changes occurring after wasp venom exposition were associated with sphingolipid, linoleic acid, and thiamine metabolism, as well as with phenylalanine, tyrosine, and tryptophan biosynthesis. These findings may provide a basis for exploring mechanisms of wasp sting sensitization and potential targets for sensitive therapy.

The effect of bee venom on the metabolic profile was also confirmed in a study on rats [75]. The research results obtained using nuclear magnetic resonance (NMR) showed that the bioactivities of bee venom are correlated to serum compounds involved in oxidation reduction or inflammatory reactions.

The development of modern, high-throughput analytical techniques (proteomics and metabolomics platforms) allow for a detailed analysis of alterations accompanying *Hymenoptera* venom allergy at the molecular level and gives a broader perspective on the subject. Understanding the inflammatory reaction caused by *Hymenoptera* venom may help to explain the complete mechanism of bee venom allergy. This knowledge may contribute to developing new, sensitive, and specific diagnostic methods for allergy. The improvement of the diagnostics is essential because, at the present state of knowledge, it is difficult to differentiate between allergies caused by different insect species, e.g., bees and wasps.

## 5. Diagnostic Challenge—Carbohydrate Determinants as the Cause of Cross-Reactivity in Patients Allergic to *Hymenoptera* Venom

Regarding allergy to *Hymenoptera* venom, a common diagnostic problem is the double positivity in tests for IgE antibodies specific to honeybee and wasp venom. In such patients, the true dual sensitization and possible cross-reactivity of venom hyaluronidases and carbohydrate determinants should be taken into account [76]. The distinction between double sensitization and cross-reactivity is critical in selecting an allergen for specific immunotherapy in patients who do not recognize the culprit insect [77,78].

Cross-reacting carbohydrate determinants (CCDs) are present on glycoproteins and are widespread in plants and insect venom [79]. Thus, anti-CCD IgE antibodies may reflect sensitization to plant or *Hymenoptera* venom allergens and are an important cause of a double positive results in vitro.

**Figure 6:**
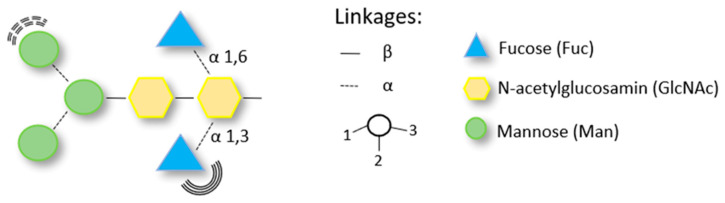
Insect venom N-glycan structure.

Many of the allergens in bee and wasp venom are glycoproteins with an N-glycan structure [80]. Major allergens such as Api m 1, Api m 2, and Ves v 2 are glycosylated. Both protein and CCD structures can act as epitopes for IgE antibodies. Glycosylation (post-translational modification of protein) occurs in the endoplasmic reticulum [80]. Plant glycoproteins are composed of asparagine (N)-linked glycans carrying the core alpha1,3-fucose and betha1,2-xylose and are represented by the two N-glycan structures: MUXF (bromelain) and MMXF (horseradish peroxidase). Insect glycoproteins contain fucose attached to the core and MMF3F6 represents their typical structure—Figure 6 [81]. The main differences between insect and plant CCDs are the presence of xylose or α1,6-fucose. IgE antibodies directed against these glycan epitopes provide the basis for in vitro cross-reactivity of allergens from various sources. Horseradish peroxidase, ascorbate oxidase, and bromelain are the glycoproteins routinely used to identify IgE reactivity to CCD determinants [80].

Research shows that up to 59% of the patients allergic to *Hymenoptera* venom reveal positive results to more than one venom. More than the half of them show specific IgE antibodies to CCDs [37]. According to data obtained by Kochuyt AM et al., IgE to CCD was detected in up to 72% of patients allergic to insects [82]. Renato Erzen et al. revealed true double sensitization in 37 patients (56.1%) and cross-reactivity in 29 of 66 participants with double positivity for wasp and bee venoms [76]. They showed that levels of anti-CCD IgE antibodies (oilseed rape OSR pollen and MUXF3) in participants with cross-reactivity were significantly higher when compared to patients with a true double sensitization to wasp and bee venoms. These results confirm that IgE antibodies against carbohydrate determinants frequently cause double positivity in in vitro tests.

The clinical relevance of anti-CCD IgE antibodies is controversial; however, it is generally believed that they are clinically insignificant [83]. Van der Veen et al. examined patients with IgE antibodies to peanuts and found that participants without a clinical peanut allergy needed a much higher concentration of peanut extract to cause basophil degranulation than clinically allergic patients [84]. Similarly, Mari et al. determined that 82.7% of patients with IgE antibodies specific to plant glycans showed IgE binding to transgenic lactoferrin. However, compared to the main grass pollen allergen, the levels of lactoferrin needed for IgE biological activity were 5–6 orders of magnitude higher [85]. In beekeepers, CCD-specific IgE antibodies were detected in only 7.7% due to a tolerance to CCD. Mertens et al. revealed the activity of anti-CCD IgE antibodies and suggested that they are clinically irrelevant in the case of insect venoms. A BAT for bee/wasp venom in participants with monosensitized *Hymenoptera* venom was positive for both venoms in 67% of patients with IgE antibodies to CCD. When using deglycosylated venom, the BAT was positive with only one insect venom [86]. However, other studies emphasized the clinical importance of CCD [87,88]. Researchers observed the degranulation of basophils in patients allergic to zucchini, celery, and tomato after exposure to bromelain, while the deglycosylated tomato allergen Lyc e 2 has lost its ability to degranulate. In addition, some participants had a positive oral challenge test with celery and zucchini; however, in vitro tests showed that these patients were only sensitized to carbohydrate determinants. Recent analysis of the natural (glycosylated) and recombinant Api m 1 bee venom allergen has shown that glycosylation increases allergenicity by presenting more epitopes. Moreover, the glycosylated allergen had the ability to more strongly induce basophil activation [89]. It should be noted that the clinical irrelevance of anti-CCD antibodies is still under discussion. It cannot be excluded that carbohydrate determinants cause clinical symptoms in some people.

The distinction between true double sensitivity to bee and wasp venom and cross-reactivity remains a challenge and makes it difficult to identify the culprit. Currently, component-resolved diagnostic (CRD) is used to distinguish between double sensitivity and cross-reactivity instead of an inhibition test. The use of advanced tools of CRD for CCD-free venom allergens enables the detailed characterization of sensitization profiles and the identification of the venom causing clinical symptoms [89,90].

## 6. Concluding Remarks

Despite developments in diagnostics of *Hymenoptera* venom allergy, new testing methodologies are still needed to answer questions and doubts we have. Proteomics and metabolomics investigations, enabling analyses of hundreds of molecules in a sample, yield a unique insight into cell status. Therefore, continuation of the performed omics studies can provide more complete information on patients’ health condition and lead to improved diagnostic capabilities. In clinical practice, there are still problems: first of all, is the distinction between asymptomatic sensitization and symptomatic venom allergy. There are many doubts in the case of cross-reactions and their differentiation with true double sensitization. Therefore, we still need the further development of already known diagnostic methods—including the introduction of new molecules using CRD, as well as completely new diagnostic methods, which may help solve the current diagnostic difficulties, and thus improve the patient’s treatment. It should be emphasized that only the correct diagnosis of allergy to *Hymenoptera* venom allows for the proper therapy of the patient, i.e., venom immunotherapy.

## Data Availability

Not applicable.
