# Correction: Matysiak et al. Diagnosis of *Hymenoptera* Venom Allergy: State of the Art, Challenges, and Perspectives. *Biomedicines* 2022, *10*, 2170

**DOI:** 10.3390/biomedicines11082224

**Published:** 2023-08-08

**Authors:** Joanna Matysiak, Eliza Matuszewska, Kacper Packi, Agnieszka Klupczyńska-Gabryszak

**Affiliations:** 1 Faculty of Health Sciences, Calisia University-Kalisz, 62-800 Kalisz, Poland; 2 Department of Inorganic and Analytical Chemistry, Poznan University of Medical Sciences, 60-806 Poznan, Poland; 3 AllerGen, Center of Personalized Medicine, 97-300 Piotrkow Trybunalski, Poland

## Error in Figure

In the original article [1], there was a mistake in Figure 2 Taxonomy of *Hymenoptera* as published. The figure shows pictures of members of the *Vespidae* family. Two of these images were wrong. *Polistes* picture showed a *Vespula*, and contrariwise *Vespula* picture presented a *Polistes*. The corrected Taxonomy of *Hymenoptera* appears below. The authors apologize for any inconvenience caused and state that the scientific conclusions are unaffected. The original article has been updated.

## Figures and Tables

**Figure 2:**
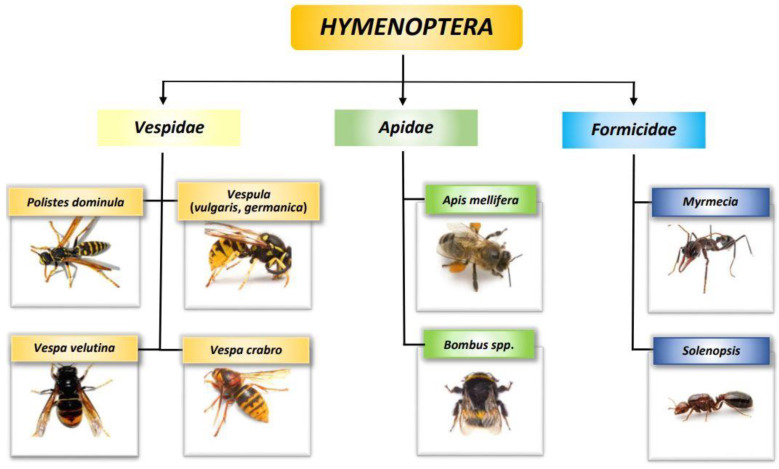
Taxonomy of *Hymenoptera*.
